# Variety-Seeking Behavior and Willingness to Pay for Staple Foods: Evidence from Online Food Delivery in China

**DOI:** 10.3390/foods15162879

**Published:** 2026-08-18

**Authors:** Meijia Li, Funing Zhong

**Affiliations:** College of Economics and Management, Nanjing Agricultural University, Nanjing 210095, China

**Keywords:** food security, diversification of staple foods, variety-seeking, willingness to pay, food delivery

## Abstract

Food security has always been the leading priority of national strategic deployment. Given the dual background of the continuous upgrading of dietary structures and the rapid growth of the food delivery industry, takeaway staple food waste has become increasingly prominent. However, a gap remains in the existing literature concerning the diversification behavior of consumers’ staple food in the context of food delivery, while even less discussion involves the driving mechanism of diversified willingness to pay (WTP). Based on 1046 survey data points related to consumption behavior concerning food and beverage takeaway staple food, this paper systematically examined the main influencing factors of consumers’ variety-seeking behavior and their WTP for staple food diversification. The following findings were obtained. There is substantial consumer demand for staple food diversification: over 80% of respondents preferred varied staple options and showed positive WTP. Dietary knowledge exerts a consistently positive and significant influence on both staple food variety-seeking behavior and consumers’ WTP for multigrain rice. An intriguing age-related pattern emerges: older consumers exhibit stronger staple food variety-seeking behavior, yet they are less willing to pay a premium for multigrain rice. In addition, dietary knowledge and frugality awareness positively affect WTP for diversified staple food portions, but personal characteristics appeared to have no significant impacts on WTP for small portions of rice. These findings provide demand-side empirical evidence and policy implications for reducing takeaway staple food waste and consolidating the foundations of food security.

## 1. Introduction

Food security is a strategic issue concerning the national economy and people’s livelihood. Data from the National Bureau of Statistics show that China’s grain output has remained stable at over 650 million tons since 2015, achieving the basic goals of grain self-sufficiency and absolute grain security. With the continuous upgrading of dietary structures and profound changes to the concept of consumption, ensuring food security in China is no longer limited to the quantity security of having enough to eat. Now, it refers more to meeting the diversified needs of urban and rural residents for better nutrition and health. In this context, it is important to distinguish between food security and nutrition security. While food security focuses on the availability and access to sufficient, safe, and nutritious food to meet basic dietary needs [1], nutrition security goes a step further, emphasizing consistent access to and consumption of foods that promote a balanced diet and meet individual nutritional requirements [2]. The policy discourse has progressively shifted from quantity-oriented food security towards quality-oriented nutrition security, which forms a more diversified and comprehensive food security system [3]. In this context, the problem of food waste has attracted increasing attention. The Anti-Food Waste Law of the People’s Republic of China, promulgated and implemented in 2021, elevated the prevention of food waste from a moral advocacy to a legal obligation, institutionalizing the role of food conservation and loss reduction within the national food security strategy. The rapid expansion of the food delivery sector has not only reshaped consumption patterns but also introduced new structural challenges for food waste management. By the end of 2024, the number of Chinese takeout users reached 545 million, while the annual transaction volume of the industry exceeded one trillion yuan [4]. Unlike traditional consumption settings such as households or restaurants, takeaway meals are typically offered as standardized set meals, affording consumers limited discretion over portion size and type. This structural constraint makes staple food waste a particularly salient issue in the food delivery context.

The food security policies in China usually focus on staple foods and rations, especially wheat and rice. The diversification of staple foods proposed in this study focuses on consumption behavior concerning traditional staple foods and the potential for replacing items like rice with non-traditional staple foods such as potato and corn. Although both of these are included in the formal definition of food, they are not included in the policy focus of food security. While food waste reduction primarily targets food loss minimization, optimizing the quality and diversity of food consumption serves as a vital complementary strategy. This relates closely to consumer variety-seeking behavior, which reflects the tendency to diversify food choices. In staple food consumption, such behavioral patterns can mitigate over-reliance on conventional staples and improve dietary quality, thereby advancing food waste reduction and nutrition security. In terms of the path to food waste reduction, the diversification of staple foods in the food delivery scenario can be understood from two dimensions. The first is portion diversification, which encourages consumers to choose appropriate sizes, such as small rice portions, which helps reduce waste. Although the amount of food saved in a single consumption scenario is limited, it will make a non-negligible marginal contribution to ensuring food security under the dual superposition effect of the large takeaway user base and the continuous growth of the takeaway industry. The second dimension is the diversification of staple food, which advocates replacing traditional staple food with non-traditional alternatives. This is of great significance to strengthening food security in the current situation, with food ration security as the core policy goal. Meanwhile, replacing traditional staple foods with tubers and other grains can also help improve food structures, reduce hidden hunger, improve food utilization, and reduce food consumption.

Previous studies have examined online food delivery [5], food waste [6], dietary diversity [7], and willingness to pay for healthy food [8]. However, at the consumption-scene level, research on takeaway food waste appears to be less extensive than in traditional dining settings, suggesting a gap that merits further attention. Most existing studies have concentrated on descriptive analyses of its current status and influencing factors. Regarding the current state of takeaway food waste, a survey by Lin et al. [9] showed that 86.5% of consumers wasted food, with 90% of them wasted more than half of their food. Zhang et al. [10] further pointed out that college students and white-collar workers were the groups with a high incidence of takeaway waste. In terms of influencing factors, Lin et al. [9] believed that aspects such as gender, cognitive attitude, the degree to which food meets expectations, and delivery services significantly impact food surpluses. Two dimensions were considered by Song et al. [11] in their analysis of takeaway waste determinants: individual subjective factors (demographic characteristics, attitudes, and consumer behavior) and takeaway supply-system factors (platform facilities and restaurant operations). Song et al. [12] drew lessons from social practice and identified four types of heterogeneous ordering subjects. They proposed that the formation mechanism of waste was mainly due to incongruity between multiple factors, such as information asymmetry, minimum delivery limits, and insufficient fit between package setting and taste.

In the field of food consumption, empirical research on variety-seeking behavior has predominantly concentrated on general food categories, with relatively little attention paid to staple food diversification within the food delivery context. In terms of consumption behavior concerning non-traditional staple foods such as cereals, Li et al. [13] determined the key factors affecting this behavior, including household income, consumer age, product price, nutritional value and taste, promotion activities, and purchase convenience. Using a choice experiment and taking millet as an example, Sha et al. [14] analyzed the influence of four attributes―organic certification, nutrition label, brand, and country of origin labeling―on the preference for and WTP for millet cereals. The study found that consumers were most willing to pay for a nutrition label, and the premium obtained by organic certification was the most significant of the segments. Moreover, Hou et al. [15] revealed that consumption intention related to cereals was negatively correlated with age but positively correlated with factors such as product processing, healthcare knowledge, promotion methods, and purchase convenience. The study by Hu et al. [16] in Northeast China added new evidence, showing that certification labels, brands, nutrition labels, and price attributes significantly affect consumer preferences. Consumers’ WTP for certification labels had the strongest influence, followed by brand.

Previous studies have several limitations. Most have concentrated on descriptive analyses of current status and influencing factors, while behavioral mechanisms and contextual drivers remain relatively underexplored. In particular, the driving mechanisms underlying consumers ‘willingness to pay for diversified staple food types and portion sizes have received even less scholarly attention. Drawing on 1046 consumer survey data points, this paper systematically examines the main influencing factors of consumers’ seeking behavior and WTP for the diversification of takeaway staples, thereby revealing their actual demand for the diversification of staple foods and addressing a gap in the existing literature. This would provide demand-side empirical evidence and policy implications for reducing takeaway staple food waste and consolidating the foundations of food security. Specifically, this paper aimed to answer the following questions: Do consumers display the behavior of seeking diversification in staple foods? What are the driving factors? Are consumers willing to pay a premium for the diversification of staples? What key factors affect the formation of consumers’ WTP?

This study contributes to the literature in several ways. From a research perspective and based on 1046 first-hand survey data points, this paper systematically examines the internal driving mechanism of consumers’ variety-seeking behavior concerning staple food. It reveals the main influencing factors of consumers’ WTP for diversification, bridges a data gap, and provides demand-side empirical evidence for the design of food waste prevention policies. In terms of the research content, the diversification of staple foods was divided into two dimensions―variety diversification and component diversification―to investigate the influencing factors of consumers’ WTP. Moreover, a differentiated explanatory framework of WTP was constructed by introducing the hierarchy of needs theory. The latter provided the theoretical basis and empirical support for the implementation of interventions for different consumer groups.

## 2. Theoretical Framework

**Figure 1:**
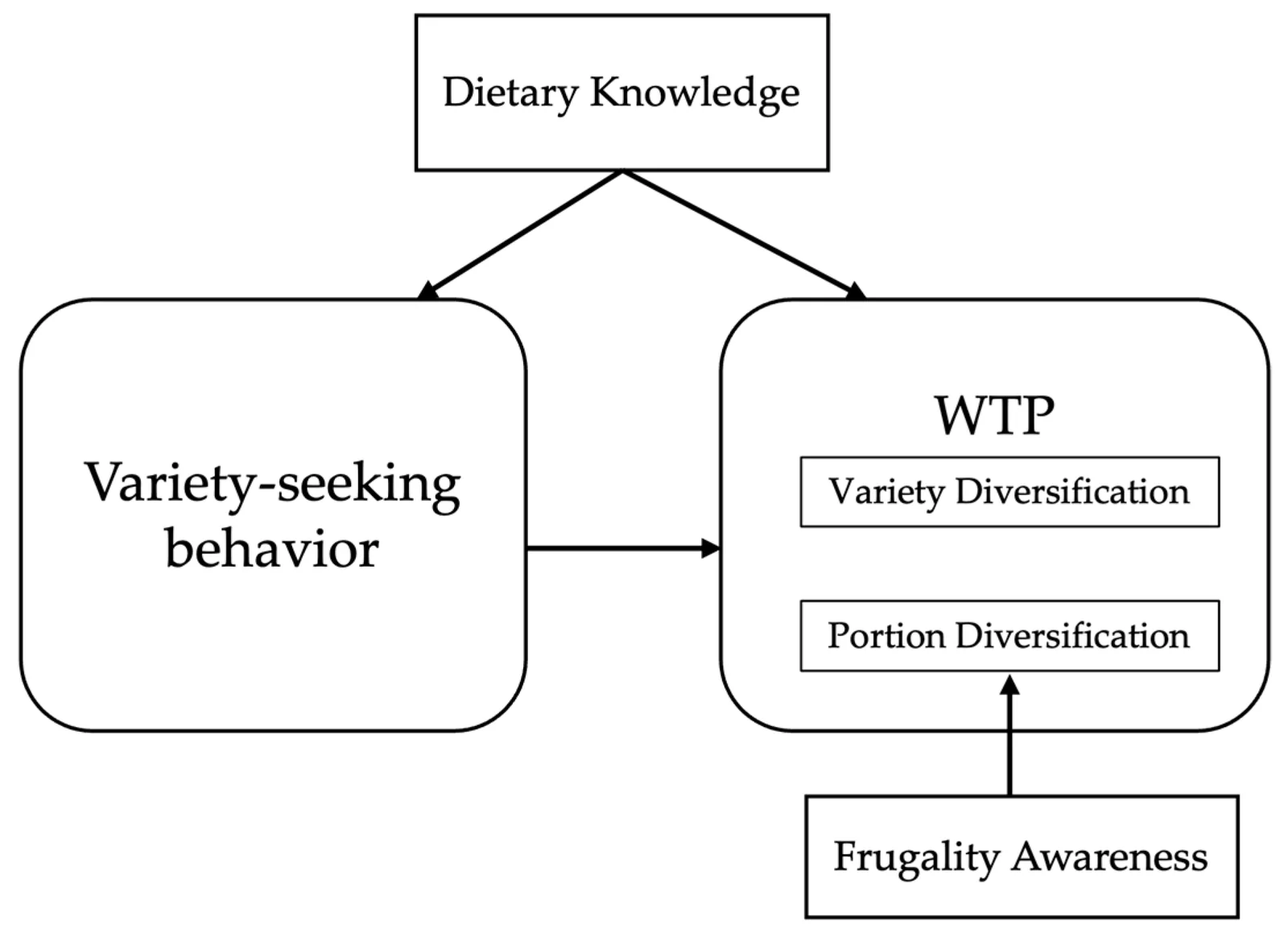
Conceptual framework of staple food diversification.

The conceptual framework of this study is shown in Figure 1. Research on variety-seeking behavior can be traced to an early study in psychology by Leuba [17], who pointed out that the internal need of individuals to seek stimulation in order to relieve boredom is the fundamental motivation of diversification behavior. On this basis, two mainstream explanations have been formed in subsequent studies. As represented by Jeuland [18], one emphasizes that consumers grow bored with a single commodity due to the pursuit of novelty, which causes a decline in marginal utility. The other, represented by McAlister [19], argues that consumers will turn to alternative products because they are tired of certain product attributes. Although these perspectives are different, both theories agree that repeated consumption of the same product will bring about utility loss. Combined with the issues explored in this study, variety-seeking means that consumers, driven by factors like internal motivation for change or external product attribute changes, tend to pursue diversity in decision-making [20].

To systematically unpack the psychological formation pathway of staple food variety-seeking behavior in online food delivery scenarios, this study adopts the Theory of Planned Behavior (TPB) proposed by Ajzen [21] as its overarching analytical framework. Widely validated in food consumption research [22], TPB posits that actual behaviors are primarily driven by behavioral intention, which is jointly shaped by three core antecedents: attitude towards the behavior, subjective norms, and perceived behavioral control. In the context of staple food choice, attitude reflects consumers’ ‘positive or negative evaluation of selecting diversified staple foods; subjective norms capture the perceived social pressure from family, peers, and prevailing dietary culture; and perceived behavioral control denotes consumers’ perceived ease or difficulty in implementing diversified staple food choices within takeaway consumption settings. Prior food nutrition studies have confirmed that dietary knowledge serves as a critical cognitive precursor reshaping the three TPB dimensions: richer nutrition literacy optimizes consumers’ attitudes toward mixed staple diets, strengthens normative perceptions of healthy eating, and lowers the perceived information and decision barriers to staple food switching, thereby elevating variety-seeking intention and subsequent actual behavior [23].

In the traditional Chinese diet, staple foods account for a relatively high proportion, but an excessive intake of these foods may lead to overweight and obesity. In this context, consumers more concerned about dietary health would be more likely to choose non-traditional staple food varieties that are more nutritious or suitable for specific needs, such as fat reduction and sugar control. From the perspective of the formation mechanism of variety-seeking behavior, the intrinsic motivation for change is a key driving force. Studies have confirmed that dietary knowledge levels affect consumers’ perceptions of food nutrition, thus changing their food choices [24]. Extensive dietary knowledge enables consumers to more accurately identify the various characteristics of different staple foods in terms of nutritional value, blood glucose response, and health efficacy. This reduces the information search cost of diversified choices and enhances their preference for variety switching. Furthermore, based on the stimulus adaptation theory [17], consumers tend to experience decreasing satisfaction with repeated consumption of the same product, while improved dietary knowledge may accelerate this adaptation process. When consumers understand the nutritional structure of different staple foods and their long-term health benefits, their motivation for change will be activated more quickly.

Embedded within the TPB framework, dietary knowledge exerts a positive influence on staple food variety-seeking: it fosters favorable attitudes towards diversified staple intake, aligns personal choices with social dietary norms, and enhances perceived control over staple switching in takeaway contexts. Consequently, consumers with higher dietary knowledge are more inclined to pursue staple food diversification on food delivery platforms, seeking both nutritional balance and an improved eating experience.

Therefore, Hypothesis 1 is proposed: the dietary knowledge level of consumers has a significantly positive impact on their staple food variety-seeking behavior.

Based on the analytical perspective of the hierarchy of needs theory, consumers display different levels of WTP for the diversification of staple foods, essentially reflecting differences in their demand for catering consumption. According to the hierarchy of needs theory, individual behavioral motivations, from low to high priority, are physiological needs, safety needs, social needs, respect needs and self-actualization needs. Complementing this, Consumer Utility Theory provides a microeconomic foundation for understanding WTP. In the canonical utility framework, consumers maximize utility from goods subject to budget constraints. In food consumption, however, utility extends beyond the satisfaction of basic physiological needs. Lombardini and Lankoski [25] posits that consumers derive utility not only from the basic attributes of a good (e.g., satiety and energy) but also from its nutritional attributes, health benefits, and the moral satisfaction associated with waste-reducing behaviors. Thus, the utility function of staple food consumption extends beyond mere calorie intake to encompass health outcomes, social identity, and ethical considerations. Integrating these theoretical perspectives with the hierarchy of needs framework, we propose a hierarchical progression of consumer demand for staple food diversification.

The primary goal of consumers with lower demand is to satisfy satiety and energy supply, and these individuals are more price-sensitive. They are less sensitive to the size of staple food and tend to choose large portions of this food, even when residual waste might be created. For them, the utility of saving waste is low, and their WTP for the diversification of staple food is mainly constrained by their income levels. With higher income levels and consumption power, consumers begin to focus on health image and social identity. Small portions of staple food carry a significant unit-price premium relative to their reduced quantity. Meanwhile, the moral satisfaction and social identity related to reducing staple food waste significantly improve the subjective utility of saving behavior. Hou et al. [26] pointed out that greater dietary knowledge would promote changes to the dietary diversity of individuals. Higher levels of this knowledge would make people realize the importance of calorie control and nutritional balance. Therefore, the willingness among such consumers to pay for the diversification of staple food items is affected by the awareness of saving and dietary knowledge level.

When it reaches a higher level, consumer demand for diversified staple foods goes beyond the basic category of satiation and health management; it becomes the pursuit of diet quality, nutritional structure, and quality of life. In terms of the variety of staple foods, although non-traditional staples such as multigrain rice, corn, and sweet potato have a higher price per unit product than traditional staple foods, their higher nutrient density and stronger satiation give them irreplaceable utility advantages in specific consumption scenarios, such as fat reduction and sugar control.

Therefore, Hypothesis 2 was proposed: The higher the dietary knowledge level of consumers, the higher their WTP for the diversification of staple foods. Both dietary knowledge and awareness of saving have significant positive impacts on the WTP for the diversification of staple food portions.

## 3. Materials and Methods

### 3.1. Data

The data used in this paper were collected through a dedicated survey that the research team conducted on the consumption behavior of takeaway staple food in four districts of Nanjing, China from July 2023 to January 2024. A stratified sampling was conducted on catering establishments that met the survey requirements, with catering establishments selected if they were takeaway businesses providing rice as the main food. Ultimately, 10 catering takeaway establishments were selected from each district, totaling 40 outlets, and at least 25 valid questionnaires were collected from each restaurant. A pilot test was conducted prior to the formal survey to assess the readability, logical flow, and completion time of the questionnaire. Based on the feedback, minor wording adjustments were made to improve clarity.

Fieldwork was carried out during weekday lunch hours. Researchers visited the selected outlets and randomly distributed cards bearing QR codes linked to the online questionnaire to customers ordering single-person takeaway meals. Consumers scanned the code to complete the questionnaire. A total of 3356 questionnaires were distributed, of which 1046 were retained after data cleaning, yielding a valid response rate of 31%. Invalid responses were excluded based on three criteria: completion time below 60 s, selecting the same option for more than five consecutive items, and logically inconsistent answers.

The questionnaire covered consumers ‘staple food variety-seeking behavior; WTP; dietary knowledge levels; frugality awareness; and demographic characteristics. Table 1 presents the socio-demographic characteristics of the sample. As shown, the sample is broadly consistent with the overall structure of China ‘s online food delivery market: users aged 26–40 accounted for 64.10% of the consumer base. In addition, 74.5% of respondents were office workers engaged in light physical activity, and over 80% reported a monthly disposable income above 5000 yuan [27].

### 3.2. Econometric Model

The following models were analyzed to explore the influencing factors of consumers’ seeking behavior and WTP for diversified staple food types when having food delivered:

(1) $$y_{1}=\lambda_{0}+\lambda_{1}{nutrition}_{i}+\lambda_{2}X_{i}+\varepsilon_{i}$$

The explained variables $y_{1}$, are consumers’ behavior related to seeking diversification of staple food and WTP for the diversification of staple food. The former variable was measured by the Shannon diversity index [28], while the latter was directly obtained by using the contingent valuation method (CVM). ${nutrition}_{i}$ is the key explanatory variable of the model, which measures the dietary knowledge levels of consumers; $X_{i}$ represents the control variables that affect consumers’ WTP for diversified staple foods. These variables include the consumers’ personal characteristics, such as their gender, age, education level, birthplace, and household per capita monthly income. $\lambda_{i}$ is the coefficient to be estimated; $\varepsilon_{i}$ is the random disturbance term.

Given that the dependent variable, consumers’ WTP for staple food diversification, is a left-censored bounded variable, we employ the Tobit model, originally proposed by Tobin [29] for limited dependent variables. Widely used in food valuation research, the Tobit model accounts for the censored nature of WTP data and avoids the biased estimates that would result from applying ordinary least squares (OLS) to such data. We therefore adopt the Tobit model as our primary estimation strategy, while the OLS model is used for comparison. All statistical analyses were performed using STATA 16.0 (Stata Corp LLC., College Station, TX, USA). To facilitate the interpretation of the estimated coefficients, we calculated the marginal effects for the key explanatory variables in the Tobit model.

To study the influencing factors of consumers’ WTP for diversified staple food portions in catering takeaway, the Tobit model and OLS model were used to analyze the following econometric models:

(2) $$y_{2}=\delta_{0}+\delta_{1}{nutrition}_{i}+\delta_{2}{frugality}_{i}+\delta_{3}X_{i}+\varepsilon_{i}$$

The explained variable $y_{2}$, is consumers’ WTP for the diversified staple food during food delivery, which is measured using the contingent valuation method (CVM). Specifically, respondents were presented with a realistic consumption scenario and were directly asked to state their WTP. ${nutrition}_{i}$ and ${frugality}_{i}$ are the key explanatory variables of the model, which measure consumers’ dietary knowledge level and self-rated frugality level. $X_{i}$ represents the control variables that affect consumers’ WTP for diversified staple foods, including variables related to consumers’ personal characteristics, such as their gender, age, and education level, as listed above. $\delta_{i}$ is the coefficient to be estimated; $\varepsilon_{i}$ is the random disturbance term.

### 3.3. Variable Measurement

The main dependent variables in this paper include consumers’ behavior of seeking diversification of staple foods, WTP for the diversification of staple food types, and WTP for the diversification of staple food portions.

To capture consumers’ staple food variety-seeking behavior, a hypothetical scenario was presented: each respondent were asked to choose one staple food from five categories (white rice, multigrain rice, corn, sweet potato, and wheat-based staple foods)—once per day over five consecutive days, with repetition permitted. Their staple food variety-seeking behavior was measured using the Shannon diversity index ($H^{\prime }$), which accounts for both the richness (the number of staple food types selected) and evenness (the relative frequency of each type) of consumers’ staple food choices [30]. For each consumer, the index was calculated as:

(3) $$H^{\prime }=-\sum_{i=1}^{S}\left(p_{i}\times lnp_{i}\right)$$

where *S* is the total number of staple food types selected by the consumer over the observation period, and $p_{i}$ is the proportion of orders for the *i*-th staple food type relative to the consumer’s total number of orders. A higher $H^{\prime }$ value indicates a greater degree of staple food diversification and stronger variety-seeking behavior.

To examine consumers’ willingness to pay (WTP) for diversified staple foods, this study focused on multigrain rice as a representative non-traditional staple, alongside white rice, corn, and sweet potato. Multigrain rice was selected because it offers a more standardized reference for measuring and comparing WTP across respondents. The contingent valuation method (CVM) was used to provide consumers with a specific consumption situation and direct questions [31]. Specifically, their WTP for a variety of staple foods was measured using the following specific questionnaire item: “If a takeaway provider can provide multigrain rice in addition to white rice, and the usual price of a portion of white rice is 2 yuan, what is the highest price you would pay for a portion of multigrain rice containing the same amount?” The question was designed in an open-ended format, allowing respondents to freely specify any positive monetary amount without being constrained by pre-set price brackets. This approach minimizes anchoring effects and reduces protest zeros, thereby providing a more accurate reflection of respondents’ true willingness to pay.

To examine consumers’ willingness to pay (WTP) for diversified staple food portions, the contingent valuation method (CVM) was employed. Respondents were presented with a realistic consumption scenario and directly asked to state their maximum WTP. Although CVM is subject to certain inherent limitations (e.g., hypothetical bias), the WTP values elicited after respondents considered their usual eating habits, product usage contexts, and personal budget constraints offer meaningful insights into consumers’ valuation of portion-level diversification. More specifically, consumers’ WTP for diversified staples was measured using the following specific questionnaire item: “If a whole box of rice sold by a takeaway provider costs 2 yuan, you will be full if you eat half of it. What is the highest price you would pay for half a box of rice?”

Consumers’ dietary knowledge level is the key explanatory variable in this paper. The dietary knowledge items were drawn from the validated China Health and Nutrition Survey (CHNS) dietary knowledge module [32], a nationally representative public dataset widely used in Chinese social-science research on nutrition. To ensure survey feasibility, we selected six CHNS items specifically related to dietary diversity and staple food choices, while retaining the original wording and format. Consistent with prior CHNS-based studies, we constructed a summated total score across the six items and used it as the explanatory variable in our regression models [33]. Each item was rated on a five-point Likert scale (1 = strongly disagree, 2 = disagree, 3 = neutral, 4 = agree, 5 = strongly agree), following the scoring scheme of He et al. [34]. The total score across the six items served as a composite indicator of dietary knowledge, with higher scores reflecting greater dietary knowledge. For items 4, 5, and 6, which were negatively worded, responses were reverse-coded during data processing to ensure consistency in score interpretation. Table 2 presents the correct response rates and score distribution for each of the six items. Among them, the highest correct recognition rate was observed for the statement “The heavier you are, the healthier you are,” whereas the lowest rate was found for “Consuming large amounts of meat products (e.g., fish, poultry, eggs, and lean meat) every day is beneficial to health,” indicating that respondents struggled to make an accurate overall judgment on this item.

Consumers’ self-rated frugality level is another key explanatory variable. Unlike previous studies, which used consumer’ knowledge of the “Clean Plate Campaign” (a national initiative aimed at reducing food waste [35]), attitudes to food waste, or attitudes to wasteful food packaging, this was measured in this study by asking a specific question in the questionnaire: “Do you consider yourself a thrifty person compared to most people you know?” Responses were recorded on a five-point scale (1 = not at all thrifty; 5 = very thrifty), with higher scores indicating greater levels of frugality.

Individual and family characteristics of consumers, including their gender, age, education level, birthplace, and household per capita monthly income, also affected the diversified seeking behavior of consumers and their diversified WTP. The definitions and descriptive statistics of all variables are presented in Table 3.

## 4. Results

### 4.1. Descriptive Analysis

1. Consumers’ WTP and price premium for staple food variety

In this study, consumers’ WTP for a given portion of multigrain rice was elicited using the typical price of white rice (2 yuan per portion) as a reference. According to the survey data, consumers quoted an average of 2.87 yuan for the same portion of multigrain rice, ranging from a minimum of 1 yuan to a maximum of 10 yuan. The premium ranged from an average of 43.5% to the highest of 400%.

Descriptive statistics for the multigrain rice premium are presented in Table 4. Consumers displayed a great acceptance of multigrain rice provided by catering takeaways, with over 80% being willing to pay the premium. Despite the large gap between the prices of small portions of rice, the price distribution is relatively concentrated. Specifically, consumers willing to pay a premium above 50% accounted for about 60% of the sample.

2. Consumers’ WTP and price premium for staple food portion size diversification

In this study, consumers’ WTP for a half-portion of rice was elicited using the full-portion price of 2 yuan as a reference. According to the survey data, consumers quoted 1.32 yuan on average as the price of a small portion of rice, ranging from 1 yuan to 4 yuan. This corresponds to an average premium of 28%, with the highest reaching 300%.

Descriptive statistics for the small-portion rice premium are presented in Table 5. Consumers displayed a great acceptance of small portions of rice provided by food and beverage takeaways, with over 50% of consumers willing to pay the premium. Notably, approximately 40% of respondents were willing to accept a premium above 30%.

### 4.2. Empirical Results Analysis

#### 4.2.1. Determinants of Consumers’ Staple Food Variety-Seeking Behavior

Table 6 reports the parameter estimation results of econometric model (1), in which the explained variable is the staple food variety-seeking behavior of consumers. Given that the dependent variable is a bounded variable, we employed the Tobit model as our primary estimation method. For comparison, we also present the OLS estimates alongside the Tobit results in Table 5. The likelihood-ratio test for the Tobit model is significant (LR χ^2^ = 105.04, *p* < 0.001), confirming the overall validity of the model. Notably, the direction and significance of the key explanatory variables are consistent across both specifications, supporting the robustness of our findings.

Column (1) reports the marginal effects of the Tobit model. The coefficient for dietary knowledge is positive and statistically significant at the 1% level. Therefore, consumers with higher dietary knowledge levels tend to exhibit more pronounced staple food variety-seeking behavior, which aligned with the theoretical expectation. This finding aligns with previous studies showing that nutritional knowledge enhances consumers’ ability and motivation to diversify their food choices [36].

Of the control variables, gender was significantly positive and significant at the 1% level (*p* < 0.01), indicating that compared with males, the staple food variety-seeking behavior of females was more significant. This finding is consistent with Feraco et al. [37]. In contrast to our initial expectation, age is positively associated with variety-seeking behavior, indicating that older consumers in our sample exhibit greater staple food diversification. A plausible explanation is that older consumers may adopt a health-oriented diversification strategy—deliberately incorporating a wider range of staple foods (e.g., multigrain rice, corn, and sweet potato) to improve dietary quality and manage chronic disease risk. This aligns with Helm and Landschulze [38], who find that while novelty-seeking declines with age, within-category variety-seeking may increase among older adults in immediate consumption contexts.

#### 4.2.2. Determinants of Consumers’ WTP for Staple Food Diversification

Table 7 reports the parameter estimation results of econometric model (1), in which the explained variable is consumers’ WTP for the diversification of staple food types. The Tobit model is statistically significant (LR χ^2^ = 36.8, *p* < 0.001), and marginal effects are reported in Column (1). The key results are robust across Tobit and OLS specifications.

The coefficient of consumers’ dietary knowledge level was positive and significant at the 1% level, indicating that greater dietary knowledge is associated with higher WTP for multigrain rice [39]. Regarding personal characteristics, age was negative and significant at the 1% level, indicating that older consumers displayed less WTP for the multigrain rice provided by takeaway food. Although older consumers acknowledge the health benefits of whole grains, they tend to be more price-sensitive, and their health-related preferences do not fully translate into monetary WTP. In contrast, younger consumers may derive greater utility from multigrain rice and are thus more willing to pay a premium. Education level was negatively significant at the 5% level. However, this result should be interpreted with caution, as the sample predominantly consists of highly educated respondents (e.g., white-collar workers and college students), with limited representation of lower-education groups.

Table 8 reports the parameter estimation results of econometric model (2). This is related to the analysis of the influencing factors of consumers’ WTP for the diversification of staple food components.

The Tobit model is statistically significant (LR χ^2^ = 18.18, *p* < 0.001), and marginal effects are reported in Column (1). The key results are robust across Tobit and OLS specifications. The coefficient of the consumers’ dietary knowledge score was positive and significant at the 10% level. This indicated that the more knowledgeable consumers were about dietary knowledge, the higher their WTP for small portions of rice. They were also more willing to pay a premium for the diversification of food staple portions. The degree of consumer saving was positive and significant at the 10% level, indicating that the positive utility created by consumer saving was greater than the disutility brought by waste. However, there was no evidence that personal characteristics had a significant impact on the WTP for small portions of takeaway rice.

#### 4.2.3. Robustness Test

Compared with plain white rice, multigrain rice has higher nutrient density, stronger satiety, and better metabolic effects. It is also more suitable for people aiming at fat reduction and sugar control. The more consumers knew about its nutritional content, the more willing they were to pay a premium for multigrain rice provided by food and beverage take-out operators. Therefore, the comprehensive indicator of dietary knowledge level was replaced by the indicator of nutritional knowledge level to test the robustness of the regression results. In terms of the measurement of nutrition knowledge, the following question was set in the questionnaire: “When buying packaged food, do you read the nutrition list?” The questionnaire was divided into five frequency levels, from 1 to 5. The higher the level, the greater the consumers’ knowledge of food nutrition content. Table 9 shows the robustness test results for the influencing factors of consumers’ WTP for the diversification of staple foods. The Tobit model is statistically significant (LR χ^2^ = 21.9, *p* < 0.001), and marginal effects are reported. Knowledge of nutrition content was positively significant at the 1% level, which is generally consistent with the benchmark regression results.

The dietary knowledge level is a comprehensive index based on the six designed questions and listed in the dietary knowledge table of the CHNS questionnaire. These questions examined consumers’ understanding of dietary structure, nutritional balance, calorie intake, and nutritional control, which was found to be relatively comprehensive. The oversupply of takeaway staple food mainly leads to excessive calorie intake. Therefore, consumers’ knowledge of calorie intake affected their WTP for small portions of rice. To address this, the questionnaire was designed with a further specific question: “Do you know which contains more calories: 100 g of steamed bread or 100 g of rice?” If the answer was correct, the consumer had a relatively good knowledge of calorie intake, while a wrong answer indicated poor knowledge of calorie intake. Table 10 shows the regression results after the key explanatory variables had been replaced. The Tobit model is statistically significant (LR χ^2^ = 12.14, *p* < 0.1), and marginal effects are reported in. The more knowledgeable consumers were about calorie intake, the higher their WTP for the diversification of staple food portions.

## 5. Discussion

Consistent with existing nutrition and consumer behavior literature, this study confirms that dietary knowledge positively promotes consumers’ staple food variety-seeking behavior. Individuals with higher nutritional knowledge tend to pursue more balanced nutritional intake and actively diversify their daily food choices to avoid a monotonous dietary structure [40]. This knowledge-driven mechanism further explains why nutritional cognition serves as a stable predictor of dietary diversity in Chinese residents’ food consumption. Reducing staple food waste and promoting dietary diversification are closely linked to resource efficiency and waste valorization. Food waste not only represents a loss of edible resources but also embodies the squandering of land, water, energy, and labor inputs throughout the food supply chain. A growing body of literature emphasizes the role of dietary diversification in reducing pressure on agricultural systems and improving resource use efficiency [41]. By encouraging consumers to shift from over-reliance on single staples to a more varied diet, food waste can be reduced at the consumption stage, while simultaneously enhancing nutritional quality and supporting the transition towards a circular food economy.

An interesting pattern emerges when comparing the age effects across our two dependent variables. While age is positively associated with staple food variety-seeking, it is negatively associated with WTP for multigrain rice. This divergence is not contradictory but rather reflects distinct underlying mechanisms. Older consumers’ variety-seeking may be driven by health-oriented dietary diversification, whereas their lower WTP reflects higher price sensitivity and a stronger reference-price effect for staple foods. Importantly, our variety-seeking indicator (the Shannon diversity index) captures within-category diversification among familiar staple foods, not willingness to try novel foods. This pattern is consistent with a previous study: older adults tend to show greater variety-seeking behavior for familiar food options in immediate consumption contexts [38]. This implies that health-driven dietary diversity does not necessarily lead to increased monetary WTP among older consumers.

From a food science perspective, the promotion of staple food diversification, particularly the shift from refined white rice to multigrain options, carries substantial nutritional implications. Multigrain rice typically contains a higher proportion of dietary fiber, resistant starch, and micronutrients compared with polished white rice [42]. These components are associated with a lower glycemic index (GI), which may help modulate postprandial glucose response and improve glycemic control [43]. In addition, increased intake of whole grains has been linked to a reduced risk of chronic diseases, including type 2 diabetes, cardiovascular disease, and certain cancers [44]. The dietary fiber content also contributes to increased satiety, which may further support weight management and reduce overall energy intake. By integrating consumer economics with nutritional science, our study contributes to a more holistic understanding of how behavioral interventions can promote dietary quality in digital food environments.

The dietary knowledge scale used in this study warrants discussion. Cronbach ‘s α for the six-item measure was 0.509, indicating relatively low internal consistency. The Kaiser-Meyer-Olkin (KMO) statistic was 0.59, below the conventional threshold of 0.6 for factor analysis. Therefore, exploratory factor analysis was not performed in this study. Additionally, the CHNS dietary knowledge module was not designed as a unidimensional scale, it covers heterogeneous nutrition topics, and lower α is expected when items are reduced—a well-known statistical property. This interpretation is consistent with the CHEI case, where the index’s multidimensionality—rather than measurement invalidity—explains its low α [45]. Given our use of a well-established, nationally representative instrument, we believe the CHNS-based content validity justifies the use of these items.

Several limitations should be acknowledged. Our sample was drawn exclusively from four central urban districts of Nanjing via QR codes placed in selected takeaway restaurants. While Nanjing is a major city with a well-developed online food delivery market, the findings may not be generalizable to rural areas or regions with different dietary cultures. The QR-code-based recruitment method may have introduced selection bias towards younger, digitally active consumers. However, this demographic skew aligns with the inherent age structure of China’s online food delivery market, suggesting that our sample reflects the primary consumer segment in this sector. The Contingent Valuation Method (CVM) used to elicit WTP is subject to hypothetical and strategic bias. We mitigated these by emphasizing the academic purpose of the study, ensuring anonymity, and using an open-ended WTP question without pre-set price brackets to minimize anchoring effects. Although respondents were required to specify a positive amount to reduce protest responses, some may still have reported extremely low values, which could affect our estimates. The low R^2^ values in both OLS and Tobit models are not uncommon in cross-sectional behavioral studies, reflecting the inherent complexity of food choice behavior. Despite this, the Tobit model is statistically significant overall, and the key predictors remain robust across specifications.

Several directions for future research emerge from this study. Future studies could adopt multi-region or nationally representative sampling to test the external validity of our conclusions. Experimental designs could address the limitations of cross-sectional data and provide more rigorous empirical evidence for the relationships between dietary knowledge, variety-seeking, and WTP. Richer measures of contextual and psychological variables would improve model fit and explanatory power. Longitudinal studies tracking behavioral changes over time would offer valuable insights into the dynamics of staple food diversification and its long-term contributions to food waste reduction and nutrition security.

## 6. Conclusions

Grounded in the strategic goal of food security in China and drawing on a survey dataset of 1046 consumers, this paper systematically discusses the main influencing factors of consumers’ variety-seeking behavior and WTP for the diversification of food staples in takeaways. It also reveals a tangible consumer demand for staple food diversification, thereby providing demand-side evidence to support the reduction of staple food waste in takeaways and the strengthening of food security. The main conclusions of this study are as follows. There is a clear and sizable market demand for staple food diversification: over 80% of respondents expressed a preference for varied staple options and demonstrated a positive WTP, signaling a commercially viable pathway for food delivery platforms to differentiate their offerings. Dietary knowledge exerts a consistently positive and significant influence on both staple food variety-seeking behavior and consumers’ WTP for multigrain rice. An intriguing age-related pattern emerges: older consumers exhibit stronger staple food variety-seeking behavior, yet they are less willing to pay a premium for multigrain rice, reflecting higher price sensitivity and stronger reference-price effects for staple foods. Both dietary knowledge and frugality awareness positively affect WTP for the diversification of takeaway staple food portions. However, there was no evidence that personal characteristics had a significant impact on the WTP for small portions of takeaway rice.

Based on these research conclusions, several policy recommendations are proposed. Dietary diversity should be incorporated into the national nutrition and health education system. Moreover, it would be productive to promote public awareness and acceptance of alternative staple foods such as whole grains through multi-channel, multi-form public education. Meanwhile, considering that consumers’ knowledge of calorie intake and nutrition control appears to be relatively weak, it is necessary to promote and popularize differentiated nutrition knowledge according to the needs of different groups. Furthermore, the public’s scientific cognition of reasonable dietary structures should be systematically improved.

Cooperation should be promoted between takeaway platforms and catering merchants to optimize the supply structure of staple food products; encourage merchants to enrich diversified staple food options like multigrain rice, corn, and sweet potato; and, via nutrition information labeling, reduce the search and decision-making costs of consumers’ diversified choices. Moreover, given the stronger diversified preferences shown by female and young consumers, it is suggested to implement precise marketing intervention strategies to effectively release the potential diversified consumption demand of staple foods.

Extensive anti-food waste publicity and education activities should be undertaken to gradually reverse consumers’ inertial consumption psychology that larger portions are more cost-effective. Such measures would also guide consumers to order food on demand and consume rationally. On the supply side, food delivery businesses could be recommended to reasonably control the portion size of staple foods and increase light-calorie options such as small portions of rice. Further suggestions are to improve the portion pricing mechanism of takeaway staple food, use price leveraging to encourage consumers to choose small-portion products, and effectively reduce the structural waste caused by an excessive supply of staple food.

## Figures and Tables

**Table 1:** Socio-demographic characteristics of respondents.

Characteristics	Group	Frequency	Percentage (%)
Gender	Male	503	48.09
	Female	543	51.91
Age	15–25	464	44.36
	26–35	447	42.74
	36–45	115	10.99
	Over 45	20	1.91
Labor intensity	Sedentary	713	68.17
	Light	131	12.52
	Moderate	202	19.31
Per capita monthly household income	Below 4000 yuan	76	7.27
	4001–8000 yuan	423	40.44
	8001–12,000 yuan	309	29.54
	12,001–16,000 yuan	99	9.46
	Above 16,000 yuan	139	13.29

Note: Monetary values are reported in RMB, USD equivalents are based on the survey period average exchange rate of 7.17 RMB/USD (PBOC).

**Table 2:** Dietary Knowledge Questions and Summary Statistics.

	Questions	True or False	Score
1	A diet with lots of fruits and vegetables is very good for health.	T	3.96
2	A diet that includes a lot of staple foods (rice and wheat, and products containing these staples) is not healthy.	T	3.15
3	Eating different kinds of food is good for health.	T	4.24
4	Eating a lot of meat (such as fish, poultry, eggs, and lean meat) every day is good for health.	F	2.73
5	White rice and refined noodles contain more vitamins and minerals than whole grains and brown rice.	F	3.08
6	The heavier you are, the healthier you are.	F	4.48

**Table 3:** Descriptive statistics of variables.

Variable	Description	Mean	Std. Dev.
Explained variable			
Variety-seeking behavior	the Shannon diversity index	0.93	0.47
WTP for multigrain rice	Maximum WTP for multigrain rice (RMB)	2.85	1.24
WTP for small portions of rice	Maximum WTP for small portions of rice (RMB)	1.32	0.39
Key explanatory variables			
Dietary	Dietary knowledge score (range [12, 30])	21.63	2.49
Frugality	Self-rated frugality level (range [1, 5])	3.21	0.70
Control variables			
Gender	Male = 1; Female = 0	0.48	0.50
Age	age	27.93	6.75
Education	Primary school and below = 1; Junior high school = 2; Secondary school/high school = 3; College and above = 4	3.92	0.33
Birthplace	City = 1; County = 2; Township = 3; Rural = 4	2.35	1.30
Income_pc	Per capita monthly household income (range [1, 9])	5.06	2.13

Note: 1. Consumers’ self-rated frugality was assigned as follows: Extremely unfrugal = 1; Unfrugal = 2; Neutral = 3; Frugal = 4; Extremely frugal = 5. 2. The per capita monthly income of the family was assigned as follows: below 2000 yuan = 1; 2000–4000 yuan = 2; 4001–6000 yuan = 3; 6001–8000 yuan = 4; 8001–10,000 yuan = 5; 10,001–12,000 yuan = 6; 12,001–14,000 yuan = 7; 14,001–16,000 yuan = 8; More than 16,000 yuan = 9. 3. Monetary values are reported in RMB, USD equivalents are based on the survey period average exchange rate of 7.17 RMB/USD (PBOC).

**Table 4:** Summary statistics of the price premium for multigrain rice.

Premium Level	Bid Amount (RMB)	Frequency	Percentage (%)
0–30%	(2, 2.3]	19	1.82
30–50%	(2.3, 2.5]	194	18.55
Over 50%	(2.5, 10]	637	60.90

Note: Percentages are based on respondents with positive WTP; non-payers are excluded. Thus, category totals may not sum to 100%.

**Table 5:** Summary Statistics of the price premium for small rice portions.

Premium Level	Bid Amount (RMB)	Frequency	Percentage (%)
0–10%	(1, 1.1]	49	4.79
10–30%	(1.1, 1.3]	118	11.55
Over 30%	(1.3, 2]	419	41.00

Note: Percentages are based on respondents with positive WTP; non-payers are excluded. Thus, category totals may not sum to 100%.

**Table 6:** Determinants of consumers’ staple food variety-seeking behavior.

Variable	Variety-Seeking Behavior	
	Tobit	OLS
Dietary	0.027 ***	0.024 ***
	(4.277)	(4.239)
Gender	−0.257 ***	−0.229 ***
	(−8.036)	(−8.089)
Age	0.007 ***	0.006 ***
	(3.088)	(3.063)
Education	0.047	0.036
	(0.982)	(0.845)
Birthplace	−0.004	−0.003
	(−0.307)	(−0.291)
Income_pc	−0.012	−0.010
	(−1.619)	(−1.459)
Constant	0.124	0.263
	(0.525)	(1.276)
LR chi2	105.04 ***	
N	1046	1046
$R^{2}$		0.095

*** *p* < 0.01.

**Table 7:** Determinants of consumers’ WTP for staple food variety.

Variable	WTP for Multigrain Rice	
	Tobit	OLS
Dietary	0.052 ***	0.045 ***
	(4.752)	(4.540)
Gender	−0.030	−0.018
	(−0.540)	(−0.365)
Age	−0.013 ***	−0.012 ***
	(−3.248)	(−3.386)
Education	−0.195 **	−0.174 **
	(−2.399)	(−2.373)
Birthplace	−0.027	−0.025
	(−1.280)	(−1.329)
Income_pc	0.003	0.004
	(0.241)	(0.349)
Constant	2.881 ***	2.977 ***
	(7.220)	(8.282)
LR chi2	36.8 ***	
N	1046	1046
$R^{2}$		0.033

** *p* < 0.05, *** *p* < 0.01.

**Table 8:** Determinants of consumers’ WTP for staple food portion size diversification.

Variable	WTP for Small Portions of Rice	
	Tobit	OLS
Dietary	0.025 ***	0.009 *
	(3.095)	(1.839)
Frugality	0.060 **	0.034 *
	(2.068)	(1.951)
Gender	−0.043	−0.022
	(−1.033)	(−0.867)
Age	0.003	0.002
	(1.144)	(0.870)
Education	−0.040	−0.045
	(−0.647)	(−1.203)
Birthplace	−0.013	−0.007
	(−0.827)	(−0.715)
Income_pc	0.002	0.003
	(0.238)	(0.565)
Constant	0.491	1.149 ***
	(1.589)	(6.126)
LR chi2	18.18 **	
N	1046	1046
$R^{2}$		0.010

Note: * *p* < 0.1, ** *p* < 0.05, *** *p* < 0.01.

**Table 9:** Robustness checks for determinants of consumers’ willingness to pay for staple food variety.

Variable	WTP for Multigrain Rice
Nutrition knowledge	0.074 ***
	(2.760)
Control variables	control
Constant	3.696 ***
	(10.505)
LR chi2	21.9 ***
N	1046

*** *p* < 0.01.

**Table 10:** Robustness checks for determinants of consumers’ willingness to pay for staple food portion size diversification.

Variable	WTP for Small Portions of Rice
Calorie knowledge	0.061 **
	(2.214)
Frugality	0.034 *
	(1.955)
Control variables	control
Constant	1.313 ***
	(7.955)
LR chi2	12.14 *
N	1046

* *p* < 0.1, ** *p* < 0.05, *** *p* < 0.01.

## Data Availability

The original contributions presented in this study are included in the article. Further inquiries can be directed to the corresponding author.
